# A Case of Christensenella hongkongensis and Bacteroides fragilis Group Bacteremia Secondary to Diverticulitis

**DOI:** 10.7759/cureus.83008

**Published:** 2025-04-25

**Authors:** Kento Goto, Mai Suzuki, Yoshinori Kanai, Toshio Naito

**Affiliations:** 1 Department of General Medicine, Juntendo University, Tokyo, JPN

**Keywords:** 16s rrna gene sequence, anaerobic gram-positive rod, bacteremia, diabetes mellitus, enterobacteria

## Abstract

Anaerobic Gram-positive rod bacteremia is typically linked to organisms such as *Clostridium perfringens* or contamination, but can occasionally be caused by less common microorganisms. Here, we present a case of bacteremia caused by *Christensenella hongkongensis*, identified through 16S rRNA gene sequencing, in a 79-year-old man who developed a fever during hospitalization for diabetes education.

## Introduction

A 79-year-old man developed a fever during hospitalization for diabetes education. Two sets of blood cultures taken at that time revealed *Christensenella hongkongensis* (*C. hongkongensis*), an anaerobic, non-spore-forming Gram-positive rod. This organism is endemic in the human intestinal tract and is related to the *Eubacterium* spp. and *Eggerthella* spp. genera [1]. *C. hongkongensis* is an Enterobacteriaceae first isolated from blood cultures of four patients in Hong Kong and Canada in 2007. A new genus and the family Catabacteraceae were proposed based on the phylogenetic position and phenotype of the 16S rRNA gene sequence. Based on 16S rRNA gene sequencing, this species has been detected in Europe, North America, and Asia. The name *Catabacter hongkongensis* was the accepted terminology from 2014 until the species was officially renamed *C. hongkongensis* in November 2021 [2].

We report a case of *C. hongkongensis* bacteremia, secondary to diverticulitis, in which the patient had a favorable outcome following antibiotic therapy. 16S rRNA analysis was performed for species identification in this case of bacteremia caused by a difficult-to-identify Gram-positive anaerobic rod. We believe that species identification by 16S rRNA analysis is important for the evaluation of patients infected with bacterial species that are difficult to identify, such as *C. hongkongensis*.

## Case presentation

A 79-year-old man with hypertension, dyslipidemia, and type 2 diabetes mellitus was hospitalized for educational admission due to poor glycemic control. On admission, he was 154.5 cm tall, weighed 43.3 kg, and had a body mass index of 18.1 kg/m^2^. On the afternoon of hospital day 13, he developed a fever and shivering chills. No significant upper respiratory symptoms, including cough and rhinorrhea, or gastrointestinal symptoms, including abdominal pain, nausea, and diarrhea, were observed. His body temperature was 39.0°C, blood pressure was 132/66 mmHg, heart rate was 107 beats/minute, respiratory rate was 17 breaths/minute, and oxygen saturation was 96% (room air).

During the COVID-19 pandemic, physical examinations were limited to throat and chest examinations per the physician’s discretion, with no abnormalities detected. Abdominal examination was not performed.

**Figure 1:**
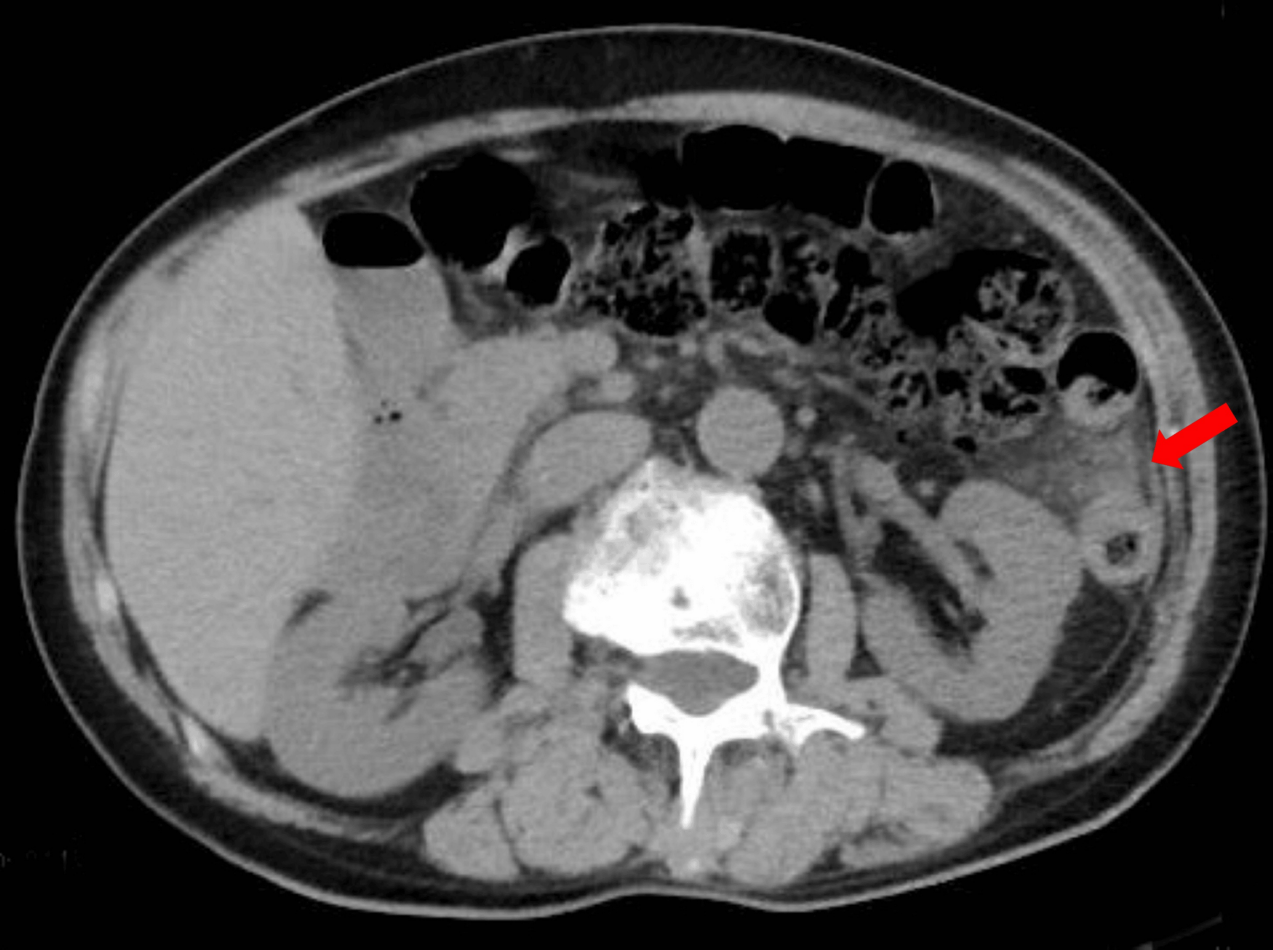
Plain CT of the abdomen. Elevated lipid concentration localized to the splenic flexure by the arrow.

Blood samples revealed the following: aspartate aminotransferase, 27 U/L; alanine aminotransferase, 16 U/L; lactase dehydrogenase, 259 U/L; blood urea nitrogen, 16 mg/dL; creatinine, 0.73 mg/dL; C-reactive protein, 0.27 mg/dL; white blood cell count, 4,700/µL (85% neutrophils); hemoglobin, 13.4 g/dL; and platelet count, 16.9 × 10^4^/µL (Table 1). Plain abdomen computed tomography (CT) showed fat stranding localized to the splenic flexure, and CT two weeks earlier showed a colonic diverticulum in the same area, leading to a suspicion of diverticulitis (Figure 1). Treatment was started with ampicillin/sulbactam 3 g every six hours.

**Table 1:** Laboratory data. AST: aspartate aminotransferase; ALT: alanine aminotransferase; LDH: lactate dehydrogenase; CRP: C-reactive protein; HbA1c: hemoglobin A1c

Labs	Values	Normal range
AST (U/L)	27	5–37
ALT (U/L)	16	6–43
LDH (U/L)	259	119–221
Urea (mg/dL)	16	19–21
Creatinine (mg/dL)	0.73	0.6–1.0
CRP (mg/dL)	0.27	<0.3
Leucocytes (/μL)	4,700	3,900–9,700
Neutrophils(%)	85	37–72
Hemoglobin (g/dL)	13.4	13.4–17.1
Platelets (×10^4^/μL)	16.9	15.3–34.6
HbA1c(%)	9	4.6–6.2

**Figure 2:**
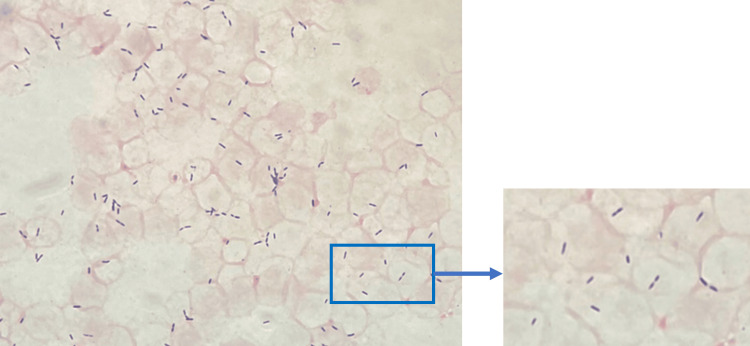
Gram-stained image of blood culture medium (anaerobic bottle).

Both sets of blood cultures were positive only in the anaerobic bottle. The time required for blood culture to become positive was 77 hours for the bottle taken from the elbow vein and 83 hours for the bottle taken from the inguinal artery. Gram staining (Bartholomew & Mittwer; Muto Pure Chemicals, Tokyo, Japan) of the bottle contents revealed small Gram-positive rods in two sets (Figure 2). Gram-negative rods were also detected in bottles containing specimens taken from the elbow vein. The species was identified as the *Bacteroides fragilis* group. The contents of the bottles were analyzed with 5% sheep blood agar/chocolate agar (Nissui Pharmaceutical, Tokyo, Japan), Drigalski lactose agar (Nissui Pharmaceutical), and Vital media Brucella HK-RS (BHK; Kyokuto Pharmaceutical Industrial Co., Tokyo, Japan). The first two media are aerobic, and BHK is anaerobic. The incubation temperature was 35°C for all cultures.

**Figure 3:**
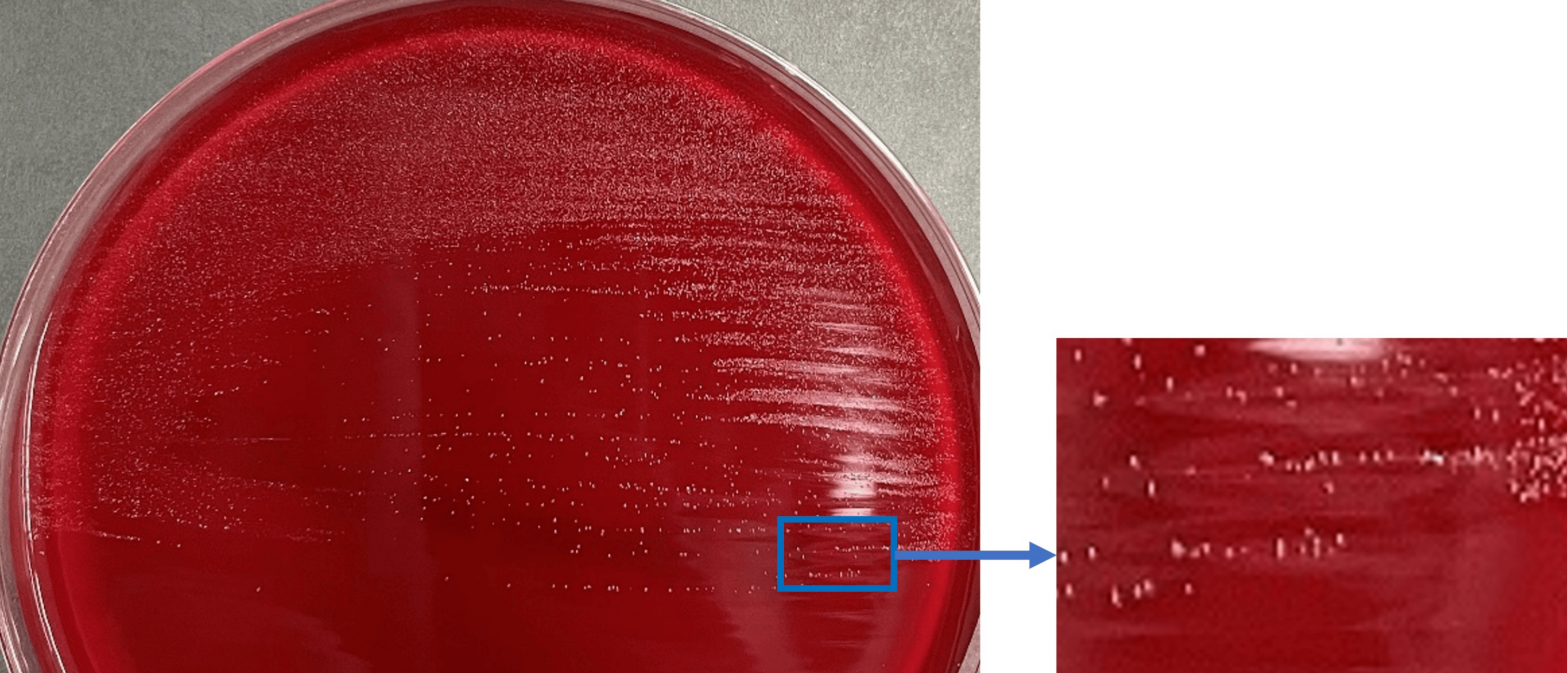
Colony characteristics of detected bacteria (Brucella HK-RS agar; four-day anaerobic culture).

No growth was observed with the aerobic media, while the anaerobic media formed grayish-white microcolonies on the second day of incubation (Figure 3). Based on the negative catalase test and morphological characteristics, we first considered the possibility of *Eggerthella* spp. Subsequently, 16S rRNA sequencing was performed and analyzed by BLAST search; 99.90%(1,046 bp/1,047 bp) homology was obtained with *C. hongkongensis*. The drug susceptibility results of *C. hongkongensis* are shown in Table 2.

**Table 2:** Results of drug susceptibility tests for isolates. ^1^: E-test; ^2^: disk diffusion method; MIC: minimum inhibitory concentration

Antimicrobial agent	MIC^1^ (µg/mL)	Diameter^2^ (mm)
Ampicillin/sulbactam	0.25/0.125	-
Ampicillin	-	24
Cefotaxime	8	-
Cefpirome	-	27
Flomoxef	-	48
Imipenem	-	48
Meropenem	≦0.002	-
Minocycline	-	28
Clindamycin	0.016	-
Levofloxacin	-	22
Vancomycin	-	20
Metronidazole	≦0.016	-

Based on these results, the patient was diagnosed with diverticulitis due to *C. hongkongensis* and the *Bacteroides fragilis* group. As the infection showed susceptibility to ampicillin according to the disk diffusion method, intravenous ampicillin/sulbactam was continued for two weeks. He also received oral amoxicillin/clavulanic acid for two weeks. He did not develop a fever subsequently. As some reports have described cases complicated with gastrointestinal malignancy, contrast-enhanced CT and upper and lower endoscopy were performed; no malignant tumors were found.

## Discussion

In diagnosing *C. hongkongensis* bacteremia in this case, blood culture results were initially reported as *Eggerthella* spp. The bacteria, which did not grow or look like typical *Eubacterium* or *Eggerthella*, showed negative results in the Rap ID ANA (Thermo Fisher Scientific). Therefore, we performed additional tests to identify the species. Genetic analysis was therefore performed, and the organism was successfully identified. The first report of *C. hongkongenesis* [3] described the organism as an anaerobic bacillus with variable motility, positive Gram staining, and catalase reaction positivity, similar to a Korean report [4]. As this species was reported to have diverse phenotypes [2], catalase reaction positivity may not always contribute to its identification.

*C. hongkongensis* is an environmental microbe that is also present in the human gut [5]. Reports of bacteremia are scarce worldwide, and most cases have been reported in patients with colon cancer and gastrointestinal and hepatobiliary infections [6]. This patient presented with bacteremia in the absence of malignant tumors or hepatobiliary disease. Furthermore, abdominal findings were unremarkable, and the patient only reported chills and general fatigue. We hypothesized that the patient’s impaired immune response, secondary to poor glycemic control associated with diabetes mellitus, contributed to the bacteremia despite the paucity of symptoms and physical findings. *C. hongkongensis* showed variable susceptibility to penicillin or cephalosporin, as this case is also resistant to cefotaxime, with inconsistent susceptibility to penicillin [7]. Therefore, metronidazole or, in cases of susceptibility, as in the present case, penicillin with a beta-lactamase inhibitor may be appropriate for treatment.

## Conclusions

We reported a rare case of bacteremia and diverticulitis caused by *C. hongkongensis* in Japan. The few published reports of *C. hongkongensis* suggest that many anaerobic Gram-positive rods in clinical laboratories are often unidentified even at the genus level, as their identification requires special equipment and expertise. We considered the application of 16S rRNA gene analysis to have the potential to reveal more strains, leading us to believe that *C. hongkongensis* bacteremia patients could be underdiagnosed. 16S rRNA gene analysis can enhance the identification of *C. hongkongensis*, with the potential of revealing a cohort of undiagnosed patients.
